# Isolation and Characterization of Bacteria from the Gut of *Bombyx mori* that Degrade Cellulose, Xylan, Pectin and Starch and Their Impact on Digestion

**DOI:** 10.1673/031.010.10701

**Published:** 2010-07-13

**Authors:** A. Alwin Prem Anand, S. John Vennison, S. Gowri Sankar, D. Immanual Gilwax Prabhu, P. Thirumalai Vasan, T. Raghuraman, C. Jerome Geoffrey, S. Ezhil Vendan

**Affiliations:** ^1^ Research Centre for Biological Sciences, Naesam Trust, Ellis Nagar, Madurai, 625016, India; ^2^ Dept. of Biotechnology, Anna University, Tiruchirappalli, 620 024, India; ^3^ Present address: Entomology Research Institute, Loyola College, Chennai, 600034, India; ^4^ Present address: University of Tübingen, Institute of Anatomy, Österbergstrasse 3, 72074 Tübingen

**Keywords:** *Aeromonas* sp., *Bacillus circulans*, *Citrobacter freundii*, *Enterobacter* sp., *Erwinia* sp., *Klebsiella pneumoniae*, *Proteus vulgaris*, *Pseudomonas aeruginosa*, *Pseudomonas fluorescens*, *Serratia liquefaciens*

## Abstract

*Bombyx mori* L. (Lepidoptera: Bombycidae) have been domesticated and widely used for silk production. It feeds on mulberry leaves. Mulberry leaves are mainly composed of pectin, xylan, cellulose and starch. Some of the digestive enzymes that degrade these carbohydrates might be produced by gut bacteria. Eleven isolates were obtained from the digestive tract of *B. mori*, including the Gram positive *Bacillus circulans* and Gram negative *Proteus vulgaris, Klebsiella pneumoniae, Escherichia coli, Citrobacter freundii, Serratia liquefaciens, Enterobacter* sp., *Pseudomonas fluorescens, P. aeruginosa, Aeromonas* sp., and *Erwinia* sp.. Three of these isolates, *P. vulgaris, K. pneumoniae, C. freundii*, were cellulolytic and xylanolytic, *P. fluorescens* and *Erwinia* sp., were pectinolytic and *K. pneumoniae* degraded starch. *Aeromonas sp*. was able to utilize the CMcellulose and xylan. *S. liquefaciens* was able to utilize three polysaccharides including CMcellulose, xylan and pectin. *B. circulans* was able to utilize all four polysaccharides with different efficacy. The gut of *B. mori* has an alkaline pH and all of the isolated bacterial strains were found to grow and degrade polysaccharides at alkaline pH. The number of cellulolytic bacteria increases with each instar.

## Introduction

*Bombyx mori* L. (Lepidoptera: Bombycidae), which feed on mulberry leaves are widely used for silk production. After hatching, the larvae begin to consume 30,000 times its own weight, of mulberry leaves and grow rapidly (Fenemore and Prakash 1992). The 1^st^ instar larvae , particularly for the first instar feed on young leaves which are rich in protein and water content. The mature instar larvae feed on mature leaves that are rich in carbohydrate with lower amounts of protein and water content (Aruga, 19 1994).

The foliage leaves are the most conspicuous organ of a plant. The structural component (primary and secondary cell wall) of leaf is composed of cellulose, xylan, pectic substance and lignin (Salisbury and Ross 2001). Mulberry leaves are mainly composed of pectin, xylan, cellulose and starch. Cellulose is the main compound in the plant cell wall. The mulberry leaves (DM basis) consists of 121 g/Kg^-1^ of cellulose and 107 g/Kg^-1^ of hemicellulose (Kandylis et al. 2009). Cellulose is a biopolymer of glucose linked by β-1, 4 glycosidic linkages (Stryer 1996). The β confirmation allows the cellulose to form a linear straight chain (Lynd et al. 1999). In most cases, cellulose fibers are embedded in a matrix of other structural biopolymer; primarily hemicellulose (xylan), pectin and lignin (Marchessault and Sundararajan 1993). Xylan consists of a backbone of β-1, 4 xylopyranose residues and it is less tightly associated than cellulose in plant cell wall (Warren 1996). Pectin is a natural structuralpolymer commonly found on middle lamella and in primary cell wall (Salisbury and Ross 2001). Pectin is composed of poly (1–4)-α-D-polygalactopyruanosyl uronic acid, in which neutral sugars are covalently bound to the polymer (Cote 1977). Starch is accumulated in chloroplast directly during photosynthesis, which is the major storage carbohydrate in plants (Jenner et al. 1982). It is composed of D-glucose connected by α-1–4 bonds and these bonds make starch chains to coil into helices (Steup et al. 1983). The composition of cellulose, xylan, pectin and starch in mulberry leaves, and the enzymes required for digestion of the above substrates along with their mechanism are summarized in Table 1.

**Table 1.**
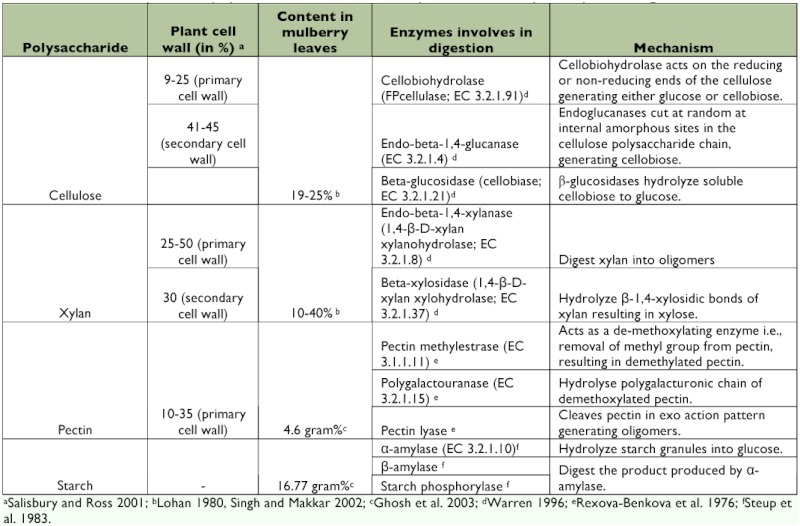
Chemical composition of polysaccharide substance in mulberry leaves and the enzymes required for digestion.

There are no specialized structures in the gut of Lepidopteran larvae, such as diverticula, and it has been assumed that microorganisms play little part in nutrition and digestion (Appel 1994; Bignell and Eggleton 1995). More recently, evidence has been presented that guts of Lepidoptera contain bacteria that produce digestive enzymes that help digestion of mulberry leaf constituents such as cellulose, xylan, pectin and starch (reviewed by Dillon and Dillon 2004). Here the hypothesis is tested that the digestive tract of *B. mori* contains bacteria that produce enzymes that digest polysaccharides including cellulose, xylan, pectin and starch that are normally difficult to digest. It is hypothesized that the nutritional contributions of gut microbiota and endosymbionts may be of several forms: 1) improved digestion efficiency, 2) improved ability to live on suboptimal diets, 3) acquisition of digestive enzymes and 4) provision of vitamins.

## Materials and Methods

### *Bombyx mori* rearing

The first instar *Bombyx mori* larvae were purchased from the Central Sericulture Research Institute, Samayanallur, South India. The larvae were reared from first to fifth instar in sterile cages at room temperature (32 ± 1°C) at a humidity of 82–90% (Upadhyayay and Mishra 2002). Larvae were fed mulberry leaves that had been sterilized by exposure to UV light. The sterilization was done in precaution to reduce external bacterial contamination. No antibiotics were used in the experiment, and none were used by the breeder. The experiments were repeated three times using separate batches of larvae purchased from the same breeder.

### Isolation and characterization of cultivatable bacteria with the property of utilizing cellulose, xylan, pectin and starch from larval digestive tract

Five *B. mori* 5^th^ instar larvae (approximately of 10 gm) were used in this experiment. The entire digestive tract was aseptically isolated in a UV laminar flow hood. The isolated digestive tract was washed with sterile icecold NaCl (0.85%) solution, chopped with a sterile blade, homogenized and incubated for 30 minutes at 37°C. The supernatant was taken and serially diluted 1000–10,000 times. The pour plate method was used to estimate total bacterial count on lysogenic broth (Bertani 2003) agar plates and on Berg's agar (Berg's et al. 1972) plates containing different substrates. The ability of the bacteria to degrade a substrate was checked using 0.1% carboxy methyl cellulose (CMC), 1% citrus pectin, 1% oat spelt xylan or 1% starch, as respective substrates. Anaerobic cultures were made to screen obligative anaerobic bacteria on these substrates. The total viable count was expressed as the number of colony forming units (CFU) in 1 ml of sample from substrate agar plates and lysogenic broth agar plates. Cellulolytic activity of cellulose-degrading bacteria in CMC medium was assayed using degradation of Whatmann No. 1 filter paper in Berg's broth (see below). As a control, a single agar plate from each batch was opened in the UV laminar flow hood for 15 minutes. This was done to check the contamination from within the hood.

### Enumeration of cultivatable total bacteria and cellulolytic bacteria from 1st to 5th instar larvae of *Bombyx mori*

The entire digestive tract was isolated from larvae of each instar for a total of approximately 10 gm, just prior to the change to the next instar. The isolation procedure was carried out as given above. The cellulose degrading bacteria were enumerated by serial dilution in Berg's agar plates containing CMC (Teather and Wood 1982), while the total bacteria were enumerated on lysogenic broth agar plates. The total viable count of cultivatable total bacteria and cellulolytic bacteria were expressed as the number of CFU in 1 ml of sample. The experiments were repeated with different batches of larvae purchased at three different times from the same breeder.

### Screening and identification of bacteria

Colonies showing degradation capacity was assayed by plate screening using the Congo red overlay method and the iodine method for each substrate (Wood 1980; Hols et al. 1994; Ruijssenaars and Hartsmans 2000). Selected isolates were plated on respective agar plates for subsequent work and maintained as pure cultures. The selected colonies with degradation capacity were identified using the Congo red overlay method and the iodine method according to Bergey's Manual of Systemic Bacteriology (Sneath et al. 1984).

For the Congo red method, plates were flooded with 0.1% aqueous Congo red for 10 minutes and then washed with 1M NaCl solution. Congo red interacts with (1–4)-β-D-glucans, (1–4)-β-D-xylan and (1–4)-a-D-polygalactopyronosyl uronic acid. A clearing zone around the colony indicates the hydrolysis of polysaccharides namely CMC, xylan and pectin respectively (Wood 1980).

For the iodine method starch plates were flooded with iodine solution resulting in dark blue plates with uncoloured zones where the starch had been degraded (Hols et al. 1994).

### Preparation of medium

Lysogenic broth agar was prepared using 10 g peptone, 5 gm yeast extract, 5 gm NaCl and 2% agar per liter. The pH was adjusted to 7.0 with NaOH, before adding agar to the medium and autoclaving. Isolated bacteria on plates were screened for ability to degrade various carbohydrates, using standard dyes: Congo red for cellulolytic, xylanolytic (Ruijssenaars and Hartsmans 2000) and pectinolytic activity, and iodine for amylolytic activity (Hols et al. 1994). The following ingredients were used for the preparation of Berg's agar (Berg's et al. 1972): minimal medium without changing its composition (in g/100 ml) of 0.2 gm NaNO_3_ , 0.05 gm MgSO_4_, 0.005 gm K_2_HPO_4_, 1 mg FeSO_4_, 2 mg CaCl_2_ , 0.2 mg MnSO_4_, and 2% agar. Berg's agar with 0.1% CMC, 1% oat spelt xylan, 1% citrus pectin and 0.1% starch on respective plates as carbohydrate substrates. Except agar, all other requirements of Berg's agar minimal medium were added in the preparation of Berg's broth.

### Assays for enzyme activity

Enzyme activity for cellulase (1, 4-β endoglucanase and FPcellulase), xylanase (1,4-β xylanase), pectinase (pectin methyl esterase and polygalactouranase) and α-amylase were assayed by measuring the amount of reducing sugar liberated from the respective substrate dissolved in appropriate buffer. The reducing sugar was measured by Dinitrosalicylic acid (DNS; Miller 1959).

### For cellulase assay

The substrate used for measuring 1, 4-β endoglucanase (EC 3.2.1.4) and FPcellulase (EC 3.2.1.91) was 1% CMC and Whatman filter paper No. 1 respectively, in 0.05M sodium phosphate buffer (pH 7.0) respectively. The enzyme action was arrested using DNS. The absorbance was measured at 540 nm. One enzyme unit was defined as the enzyme amount which releases 1 µM of glucose equivalent from substrate per minute.

### For xylanase assay

The substrate used for measuring 1,4-β endoxylanase (EC 3.2.1.8) was 1% oat spelt xylan in 0.05M potassium phosphate buffer (pH 6.0). The enzyme action was arrested using DNS and the absorbance measured at 540 nm. One enzyme unit was defined as the enzyme amount that released 1 µM of xylose equivalent from oat spelt xylan per minute.

### For pectinase assay

The substrate used for measuring pectin methyl esterase (EC 3.1.1.11) and polygalactouranase (EC 3.2.1.15) was 1% citrus pectin in 0.05M Sodium phosphate buffer (pH 7.0). The polygalactouranase was measured by stopping the reaction with DNS and reading the absorbance at 540nm. One enzyme unit was defined as the enzyme amount which releases 1 µM of equivalent galactouronic acid per minute. Pectin methyl esterase was analyzed by the release of methanol with the help of alcohol oxidase. The absorbance is measured at 412 nm. One enzyme unit was defined as the enzyme amount which releases 1 µM of methanol per minute.

**Table 2.**
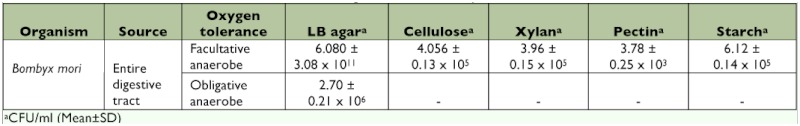
Characteristics of the bacteria isolated from the digestive tract of *Bombyx mori*.

### For amylase activity

The substrate used for studying α-amylase (EC 3.2.1.10) was 1% starch. The reaction was arrested using DNS and absorbance measured at 540nm. One enzyme unit was defined as the enzyme amount which releases 1 µM of maltose per minute from the substrate.

### Enzyme activity at different pH

The isolated bacterial strains were subjected to grow on different pH ranging from pH 4.0 10 in lysogenic broth to check its growth in alkaline pH. Selected cultivatable bacterial strains were subjected to different pH ranging from pH 4.0 - 10.0 and analyzed for FPcellulase, 1,4-β endoglucanase, 1,4-β endoxylanase, pectin methyl esterase, polygalactouranase and amylase activity. The substrates used in the experiments were as described above. The bacterial strains used in the experiment are *S. liquefaciens* for FPcellulase and 1,4-β endoglucanse, *B. circulans* for 1,4-β endoxylanase and aamylase, and *Erwinia sp.*, for pectin methyl esterase and polygalactouranase.

### Statistical analysis

Results are expressed as Mean ± SD of three replicates. They were subjected to one way ANOVA to detect statistical significance.

## Results

### Bacterial isolates from the digestive tract of *B. mori*

The total cultivatable bacterial count of the entire digestive tract was found to be 6.080 ± 3.08 × 10^11^ CFU/m1 of *B. mori* larval digestive tract suspension for cultivatable facultative anaerobic bacteria and 2.7 ± 0.21 × 10^6^ CFU/ml for cultivatable obligatory anaerobic bacteria (Table 2). Results subjected to ANOVA shows that there is statistical significance between each *B. mori* instar and cultivatable cellulose facultative anaerobic bacteria (P≥0.05). Eleven isolates were selected from the facultative bacteria and characterized biochemically. These colonies were found to be *Bacillus circulans, Proteus vulgaris, Klebsiella pneumoniae, Escherichia coli, Citrobacter freundii, Serratia liquefaciens, Entrobacter sp., Pseudomonas fluorescens, P. aeruginosa, Aeromonas sp.*, and *Erwinia sp. P. aeruginosa* and *E. coli* did not utilize any of the polysaccharide substrates used: cellulose, xylan, pectin and starch. Given its omnipresent nature, *E. coli* might have been a contaminant.

**Figure 1.**
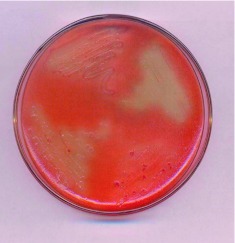
Plate showing cellulose degrading bacteria. High quality figures are available online.

No obligatory anaerobic bacteria were isolated from *B. mori* with the property to degrade cellulose, xylan, pectin or starch. The reason might be that those bacteria may not be cultivatable with the available methods. No fungal colonies were observed during the experiments. There were no colonies growing on control plates, suggesting minimal contamination from the surroundings.

### Bacterial isolates utilizing polysaccharides from the digestive tract of *B. mori*

The total cultivatable cellulose degrading bacterial count was found to be 4.056 ± 0.13 × 10^5^ CFU/ml of *B. mori* larval digestive tract suspension. From that, seven isolates were selected with cellulolytic activity. Among the seven isolated bacterial colonies, one isolate belongs to Gram-positive bacteria and other six isolates were found to be Gram-negative. The Gram-positive bacteria found to *Bacillus circulans*. The Gram-negative bacterial isolates were *Proteus vulgaris, Klebsiella pneumoniae, Enterobacter sp., Citrobacter freundii, Serratia liquefaciens* and *Aeromonas sp*. Except *Aeromonas sp.*, other bacterial isolates utilizing CMC (Figure 1) were found to utilize Whatmann No.1 filter paper in the Berg's broth which confirms that these bacterial isolates were cellulolytic bacteria.

The total cultivatable xylanolytic bacterial colonies were found to be 3.96 ± 0.15 × 10^5^ CFU/ml of the *B. mori* digestive tract suspension. The isolates utilizing xylan were found to be *B. cirulans, C. freundii, K. pneumoniae, P. vulgaris, S. liquefaciens* and *Aeromonas sp*.

The total cultivatable pectinolytic bacterial colonies were about 3.78 ± 0.25 × 10^3^ CFU/ml of the *B. mori* digestive tract suspension. *B. cirulans, Pseudomonas fluorescens* and *Erwinia sp.*, were the bacterial isolates found to be pectinolytic bacteria.

The total cultivatable starch degrading bacterial colonies were about 6.12 ± 0.14 × 10^5^ CFU/ml of the *B. mori* digestive tract suspension. The isolates utilizing starch were found to be *B. circulans, S. liquefaciens* and *K. pneumoniae*.

### Identification of bacterial isolates from *B. mori* digestive tract

The Gram-positive bacteria was identified and confirmed as *B. circulans* (Table 3). The isolated strains of Gram-negative bacteria were rod shaped. Upon biochemical classification (Bergey's Manual of Systematic Bacteriology), these isolates were confirmed to belong to the Family Enterobacteriaceae (summarized in Table 4). The isolate with morphology of straight rod was confirmed to be *Aeromonas sp.*, (Table 5). Members of the genus *Pseudomonas* was identified by their positive result for motility, indole utilization, VP, citrate utilization, glucose fermentation, oxidase reaction and nitrate reduction and negative result for methyl red and H_2_S production (summarized in Table 6).

**Table 3.**
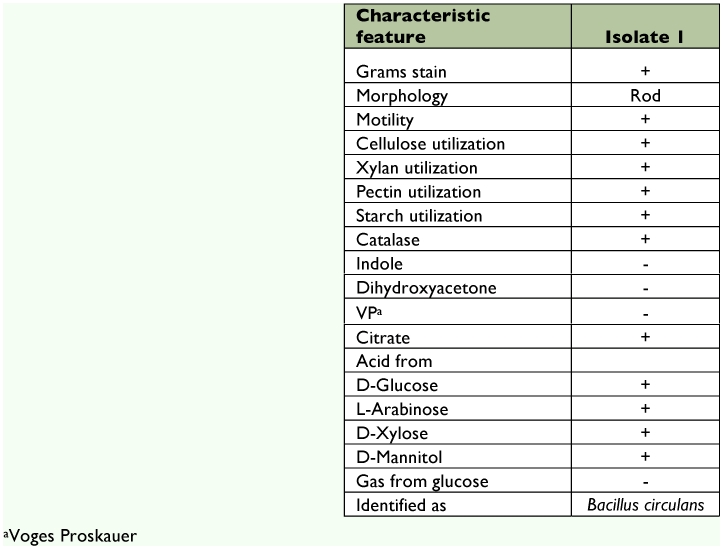
Gram-positive bacteria isolated from the digestive tract of *Bombyx mori*.

### Cellulolytic bacteria

**Figure 2.**
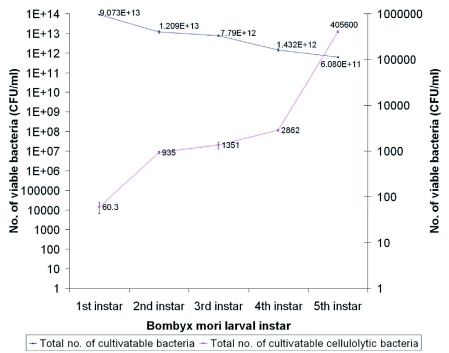
Enumeration of bacteria from the digestive tract of *Bombyx mori* with total number of bacteria and total cellulolytic bacteria with respect to the different larval stages (given in CFU/ml). High quality figures are available online.

Enumeration of cultivatable bacteria from the digestive tract shows that there was a gradual decrease in the total number of bacteria in the digestive tract (Figure 2). In contrast, there was a sharp increase in the total cellulolytic bacterial count. Both trends were found to be statistically significant (P ≥ 0.05). Using Pearson's correlation R = -0.29, there was a negative correlation between total bacterial count and total cellulolytic bacteria with respect to the growth i.e., first to fifth instar. The increase in cellulolytic bacterial count with increase in larval stage can be attributed to the increased volume of food consumed. No obligate anaerobes with the ability to degrade cellulose were found.

**Table 4.**
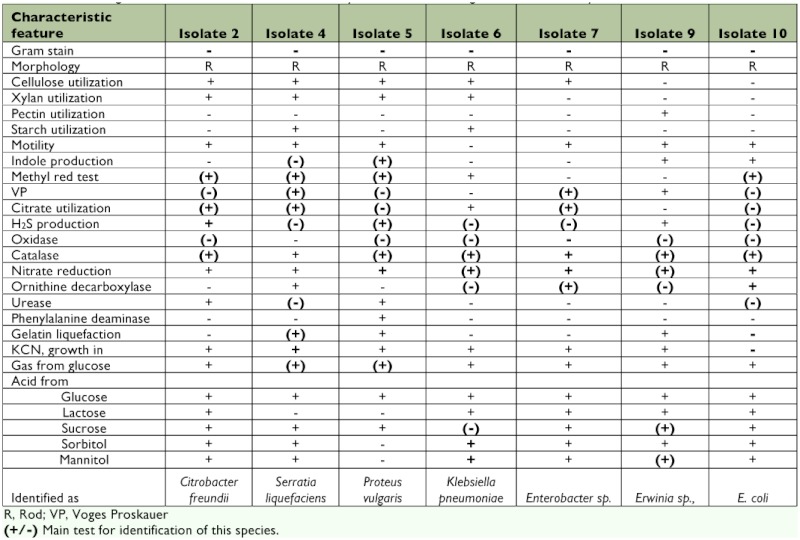
Gram-negative bacteria of *Enterobacteriaceae* family isolated from the digestive tract of *Bombyx mori*.

**Table 5.**
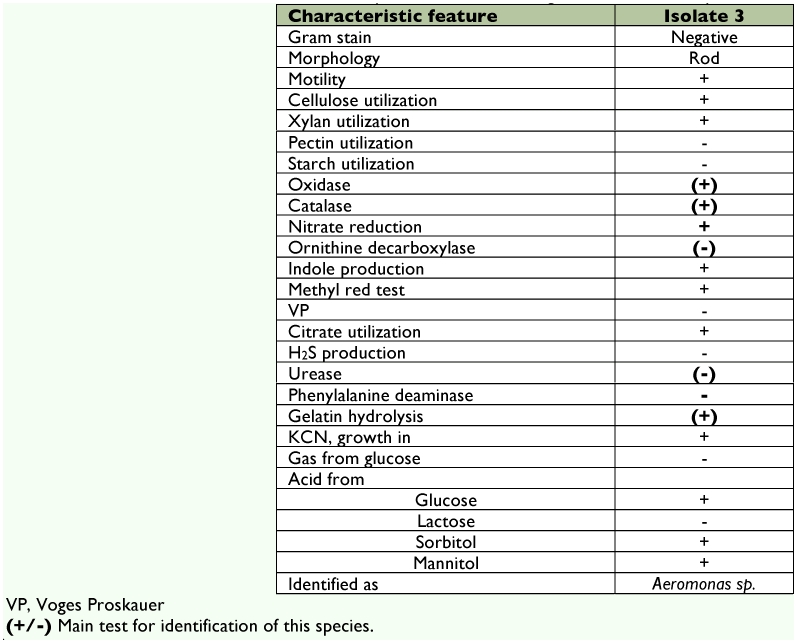
Characteristic features of *Aeromonas sp*., isolated from the digestive tract of *Bombyx mori*.

**Table 6.**
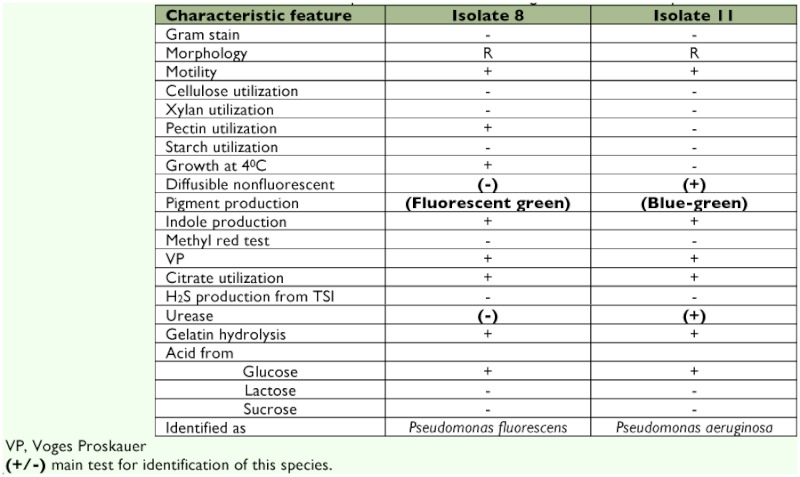
Characteristic features of *Pseudomonas* species isolated from the digestive tract of *Bombyx mori*.

### Enzyme activity of the bacterial isolates from the digestive tract of *B. mori*

**Figure 3.**
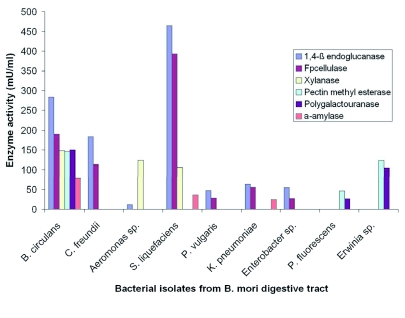
Enzyme activity of isolated and characterized bacterial strains. High quality figures are available online.

The enzyme activity of the isolated bacteria is summarized in Figure 3. The bacterial count of starch degrading bacteria was more than other substrates degrading bacteria (Table 2). *B. circulans* found to utilize all the polysaccharides and have maximum activity of starch degradation in comparison with other bacterial isolates. *C. freundii* utilize cellulose of amorphorus and of crystalline origin. *Aeromonas sp.*, has higher xylanse activity with less amorphorus cellulose degradation. *S. liquefaciens* have higher cellulolytic, xylanase and amylase activity. *P. vulgaris, K. pneumoniae* and *Enterobacter sp.*, were found to have cellulolytic activity with less efficiency compared with *S. liquefaciens*, and *B. circulans. P. flurorescens* and *Erwinia sp.*, utilize pectin at the maximum.

**Figure 4.**
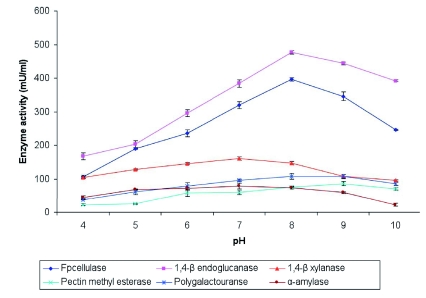
Enzyme activity of selected bacterial strains at different pH. (FPCellulase and 1,4-β endoglucanase - *Serratia liquefaciens*, 1,4-β xylanase and α-amylase - *Bacillus circulans*, pectin methyl esterase and polygalactouranase - *Erwinia sp*,). High quality figures are available online.

All bacterial isolates were able to grow on pH ranging from pH 5.0–9.0. The selected bacterial strains were able to grow on alkaline pH (Figure 4) and are capable of degrading the polysaccharide substrates at this pH. The enzyme activity peaked at pH 8.0 for FPcellulase, 1,4-β endoglucanse and polygalactouranase. 1,4-β endoxylanase and α-amylase had an optimum at pH7.0. Pectin methyl esterase had an optimum at pH 9.0. These are summarized in Figure 4.

## Discussion

We have isolated cultivatable bacteria from *B. mori* with the capability of utilizing various polysaccharides. The bacteria isolated were *Aeromonas sp., B. circulans, C. freundii, Enterobacter sp., Erwinia sp., K. pneumoniae, P. vulgaris, P. fluorescens* and *S. liquefaciens*.

Many reports have been published regarding bacteria of the digestive tract of insects. *C*. *freundii* and, *Pseudomonas sp.*, are found in the digestive tract of the ground beetle, *Poecilus chalcites* (Lehman et al. 2008). In *Aedes aegypti, Bacillus sp., Bacillus subtilis* and *Serratia sp.*, were found in the gut diverticulum (Gusmao et al. 2007). An experiment with plant epiphytic *E. herbicola* in the gut of *B. mori* showed that they were able to grow and survive in the gut (Watanabe et al. 1998a). *Aeromonas sp*. with xylanase activity was isolated from the intestine of the herbivorous insect, *Samia cynthia pryeri*, (Roy et al. 2003). *P. vulgaris, C. freundii, S. liquefaciens* and *Klebsiella sp.*, were reported to be cellulose degrading bacteria and xyalnolytic bacteria (Alwin and Sripathi 2004). Here based on the observed results, we examine the role of bacteria in the digestion of polysaccharide in mulberry leaves.

### Nutritional role of bacteria in digestion

Raman et al., (1994) observed food consumption and utilization in *B. mori* larvae and concluded that: 1) the approximate digestability (AD) and efficiency of conversion of ingested food (ECI) were inversely correlated to the larval stage. 2) ingesta and digesta required to produce lgram body weight progressively increased from the 1^st^ — 5^th^ instar. We have observed that the total cultivatable bacterial count decreased from first to fifth instar larva, while the cellulolytic bacterial count increase from first to fifth instar (Figure 2). The result is statistically significant (P ≥ 0.05) with growth from the first to fifth larval instar. The increase in cellulolytic bacteria was directly proportional to the ingesta and digesta observed by Raman et al., (1994), which shows that, there is a relationship between gut bacteria and digestion. The reduction of total bacterial count from first to fifth instar larvae might be the result of the increase in cellulolytic bacteria.

Mulberry leaves were used for the cultivation of silkworms. Leaves that were used for young, particularly first larval stage, are rich in protein and water content, but poor in carbohydrate content. As leaves grow, their protein and water content decreases and the carbohydrate content increases (Aruga 1994). The bacterial isolates, obtained from the fifth instar larvae were found to have the ability to digest cellulose, xylan, pectin and starch, all of which are found in mulberry leaves. This suggests that these bacteria may secrete enzymes important in digestion.

### Cellulase and xylanase activity

Most herbivorous and xylophagous insect intestines contain various symbiotic microorganisms that degrade biopolymers like cellulose and xylan (Mannesmann 1972). In the intestine of *Samia cynthia pryeri*, xylanase activity was found in the intestine. It is secreted by *Aeromonas* sp., (Roy et al. 2003). All the isolated cultivatable cellulolytic bacteria utilize both the forms of cellulose (amorphous and crystalline) although with different specificities (Figure 3). The majority of the cellulolytic bacteria were found to be Enterobacteriaceae. Usually cellulose degrading bacteria are suggested to have the ability to utilize xylan which is a polymer made of β-1,4 xylosidic bonds. *S. liquefaciens, C. freundii, K. pneumoniae, P. vulgaris* and *B. circulans* were found to ability to utilize xylan. *S. liquefaciens* and *B*. *circulans* were cellulolytic as well as xylanolytic bacteria and were able to grow in alkaline pH (Figure 4) which is also the gut pH, suggesting that they might play a role in digestion of cellulose and xylan in the mulberry leaves consumed by *B. mori*.

It was also observed that there is a proportional increase in cellulolytic bacteria with the growth of the *B. mori* larval instar (Figure 2). The relationship between total bacterial count and total cellulolytic bacteria was inversely proportional (Pearson's correlation R = -0.29), with respect to the growth of *B. mori*. These bacterial isolates may be passed to the next generation.

*B. mori* utilizes disaccharides, especially sucrose, cellobiose and maltose (Ito 1967). Cellulose hydrolysis requires three enzymes namely cellobiohydrolase (=FPcellulase; EC 3.2.1.91), endo-beta-1,4-glucanase (EC 3.2.1.4) and cellobiase (beta-glucosidase; EC 3.2.1.21) (Warren 1996). Cellobiohydrolase acts on the reducing or non-reducing ends of cellulose generating either glucose or cellobiose. Endoglucanase digests internal amorphous sites in the cellulose polysaccharide, releasing oligomeres of various length. Beta-glucosidase cleaves the cellobiose producing glucose (Lynd et al. 2002).

*B. mori* expresses the beta-glucosidase in the midgut. The expression was observed only during the feeding period. This enzyme belongs to Class 2, which can only hydrolyze cellobiose and lactose (Byeon et al. 2005). Beta-glucosidase is usually involved in the hydrolysis of di- and oligo-β-saccharides derived from xylan and cellulose in the diet (Terra and Ferreira 1994). We have isolated bacteria that utilize both amorphous and crystalline cellulose into glucose or cellobiose, after which endogenous betaglucosidase converts cellobiose into glucose. Glucose is assimilated in the microvillar structures in the midgut.

### Pectinase activity

Pectinase (polygalactouranase) occurs in the Orders Orthoptera, Hemiptera, Coleoptera, Diptera and Trichoptera, but so far no pectinase has been shown to be produced by an insect (Dillon and Dillon 2004). In desert millipedes *Orthoporus ornatus* and *Comachelus* sp., pectin degradation was observed and it was suggested that the pectinase might be of microbial origin (Taylor 1982). In Longicorn beetle species, pectinase producing bacteria were found and reported as a source of digestive enzyme (Park et al. 2007). Pectinase activity in Heteroptera and Hemiptera was suggested to play a role in egg laying behaviour (Boyd et al. 2002). Here we have isolated cultivatable bacteria namely *B. circulans, P. flurorescens* and *Erwinia sp.*, from fifth instar *B. mori* larvae, which utilize pectin efficiently. These bacterial strains were also able to grow in alkaline pH (Figure 4) suggesting that these bacterial strains could be involves in digestion of pectin from mulberry leaves.

### Amylase activity

Murakami (1989) suggested that efficient starch utilization in the larval stage might have adaptive significance in non-dispasuing (Indian) strains. We also found the cultivatable bacterial population of starch degrading bacteria is higher than other polysaccharide degrading bacteria in fifth instar larvae (Table 2). *B. circulans* shows higher amylase activity than other bacterial isolates with an optimum enzyme activity maximum at pH 7.0 (Figure 4). This bacterial strain could be present in the largest numbers in the digestive tract of fifth instar *B. mori* larvae and, along with endogenous amylase, be involved in the digestion of starch products.

### Enzyme activity and gut pH

In general, the pH of the forgut in most lepidopteran larvae has a pH of about 7.0, and a very alkaline midgut, which is composed of an anterior ventriculus with pH of about 9.8, a middle ventriculus with pH of about 10.0 and a posterior ventriculus with pH of about 9.5 (Terra and Ferreira 1994). Endogenous aamylase from the midgut of *B. mori* is said to function best at pH 9.3 and was found to have an action pattern similar to porcine pancreas amylase (Kanekatsu 1978; Terra and Ferriera 1994).

Larvae of *B. mori* possess the ability to hydrolyze various carbohydrates present in plant leaves, perhaps with the help of enzymes produced by bacteria. The cultivatable bacterial isolates from *B. mori* could produce enzymes capable of digesting cellulose (amorphous and crystalline), xylan, pectin and starch. The high pH of the gut might be an adaptation of leaf-eating Lepidoptera for digesting hemicellulose (Terra 1988), for which the enzymes are usually provided by microbiota. So far no endoxylanase have been reported to be produced by insects. The isolated bacterial strains were able to utilize the substrates with efficiency at alkaline pH (pH 8.0) with the exception of amylase, which shows an optimum activity at pH 7.0 (Figure 4). Lepidopterans generally have a midgut pH near 8 that is thought to be an adaptive response for the digestion of their diets (Clark 1999). The correlation between enzyme activity and gut pH suggests that the bacteria may help in utilization of the polysaccharide substrates from mulberry leaves.

Usually, endogenous enzymes play a major role in digestion. Endogenous cellulases have been reported in several insects and termites (Watanabe et al. 1998; Tokuda et al. 1999; Girard and Jouanin 1999; Lee et al. 2004). In the yellow-spotted longicorn beetle *Psacothea hilaris*, polygalactouranse, 1,4-β-endoglucanase, 1,4-β-xylanase and βglucosidase were found to be secreted into the gut (Scrivener et al. 1997). The endogenous beta-glucosidase has been cloned from the midgut of *B. mori* and was observed to have high activity at pH 6.0–7.0 (Byeon et al. 2005), irrespective of the luminal pH from where it was isolated. Similar results were reported in other species of Orthoptera, Hemiptera, Coleoptera, Diptera and Lepidoptera (see review Terra and Ferriera 1994). We can assume *in vivo* enzyme activity is entirely different from that of *in vitro* experiments. More sophisticated methods are needed to know how this enzyme functions *in vivo*, for example, by perhaps involving buffering agents from bacteria or luminal cells.

### Insect gut and microbiota

In insects, the location of enzyme in the digestive tract varies from species to species. In desert millipedes, *Orthoporus ornatus* and *Comachelus* sp. cellulose and xylan degradation was found in the midgut, while pectin degradation was found in hindgut (Taylor 1982). In *Rhynchosciara americana* larvae (Ferriera and Terra 1983) and *Spodoptera frugiperda* (Lepidoptera: Noctuidae; Marana et al. 2000), β-glucosidase (cellobiase) was observed in the midgut cells. In *Deraeocoris nebulosus*, amylase was found in the anterior midgut, α-glucosidase and pectinase found in salivary gland as well as the anterior midgut (Boyd et al. 2002). In *Diatraea saccharalis* β-glycosidases namely βGlyl, βGly2 and βGly3 were found in the midgut, in which βGly1 and βGly3 helps in the degradation of oligo and disaccharides from xylan and βGly2 helps in glycolipid hydrolysis (Azevedo et al. 2003). In *Dysdercis peruvianus* α-glucosidase are produced in perimicrovillar membranes, aminopeptidase from the perimicrovillar space and βglucosidase from microvillar membranes (Damasceno-Sa et al. 2007).

Early studies in *B. mori* revealed that disaccharidases were absent in regurgitated material, but are present in the midgut tissues (Horie 1959). In lepidopteran larva, it was proposed that initial digestion occurs in the endoperitophic space, whereas the intermediate and final digestion takes place in the midgut cells. Initial digestion includes the breakdown of complex polymer sugars into dimers or oligomers, by enzymes such as amylase, cellulase, hemicellulase and trypsin. The final digestion includes the digestion of disaccharides and oligosaccharides into monomers, by enzymes including maltase, cellobiase and dipeptidase (Terra and Ferreira 1994). Minami et al, (1991) reported that aminopeptidase, alkaline phosphatase and ATPase were found in microvilli (brush borders) in midgut cells of *B. mori*. Likewise, β-glucosidase (cellobiase) was observed in the midgut cells of *B. mori* (Byeon et al. 2005). Here we propose that most of the enzymes such as cellulases (β-endoglucanase, cellobiohydrolase/FPcellulase), xylanase and pectinase are produced from microbial origin, and enzymes including amylase and βglucosidase are produced endogenously.

Genta et al., (2006) reported that amylase, cellulase and β-glucosidase were produced by the midgut of *Tenebrio molitor* larvae treated with antibiotics to create sterile conditions compared to non-treated controls. They suggested that the microbial-derived enzymes may have an auxiliary, non-essential digestive role, which may come into play during adaptation of the insect's hosts to different diets. In the velvetbean caterpillar, *Anticarsia gemmatalis* the role of gut bacteria was said to contribute proteolytic enzymes, as a versatile adaptation to protease inhibitors in the diet (Visotto et al. 2009). Rahmathulla et al, (2006) reported that in *B. mori* which, when treated with antibiotics showed no difference in food consumption in comparison to the non-treated larvae. However, the ingesta required to produce one gram of larva including cocoon and shell, was significantly lower in the antibiotic treated group, while the efficiency of conversion for larva, cocoon and shell was not significantly different from that of control. But higher assimilation and conversion of food was observed in the antibiotic treated group. This raises the question of whether all enzymes necessary for the digestion of mulberry leaves are secreted endogenously.

Gut micro-organisms have the ability to adapt themselves to changes in insect diet, by induction of enzymes or by population changes in the microbial community (Kaufman and Klug 1991; Santo Domingo et al. 1998). It was shown in adult pigs that dietary fibres influence xylanolytic and cellulolytic bacteria, confirming the relationship between fiber-degrading bacteria and fiber digestion, which was directly proportional to the increase in fiber-degrading bacteria and fiber digestion (Varel et al. 1987). Similarly, when cockroaches were fed on a diet rich in cellulose, there was an increase in the protozoan population in the hindgut (Gijzen et al. 1994). In *B. mori*, cellulolytic bacteria increase with the growth of the larvae (Figure 2). It is possible that the increase in cellulolytic bacteria is due to the increase of cellulose or hemi-cellulose in their diet. Insects with rapid food throughput often harbour indigenous microbiota (Dillon and Dillon 2004). We are not sure whether bacteria isolated from *B. mori* were indigenous, but the cellulolytic bacteria might be of indigenous origin as they were found to be present in the first to fifth instar larva.

We suggest that bacteria provide digestive enzymes in a synergic manner and contribute to larval growth. However, it is not clear how the *in vitro* results obtained here relate to the situation *in vivo*. Furthermore, the relative roles of endogenously and exogenously produced enzymes is not clear in *B. mori*. We are currently analyzing endogenous enzyme production in *B. mori*.

## Abbreviations

CFU: colony formaing units;
CMC: carboxy methyl cellulose
